# Not myopathic, but autonomic changes in patients with long-COVID syndrome: a case series

**DOI:** 10.1007/s10072-023-06637-8

**Published:** 2023-02-03

**Authors:** Tommaso Bocci, Alessandro Bertini, Laura Campiglio, Stefano Botta, Giorgia Libelli, Matteo Guidetti, Alberto Priori

**Affiliations:** 1 ’’Aldo Ravelli’’ Center for Neurotechnology and Experimental Brain Therapeutics, Department of Health Sciences, University of Milan, Via Antonio di Rudinì 8, 20142 Milan, Italy; 2 ’’Aldo Ravelli’’ Center for Neurotechnology and Experimental Brain Therapeutics, Department of Health Sciences, University of Milan, Via Antonio di Rudinì 8, 20100 Milano, Italy; 3 Clinical Neurology Unit, “Azienda Socio-Sanitaria Territoriale Santi Paolo E Carlo” and Department of Health Sciences, University of Milan, Via Antonio Di Rudinì 8, 20142 Milan, Italy; 4 Department of Medicine and Surgery, Neurology Unit, University of Parma, Via Gramsci 14, 43126 Parma, Italy; 5 Department of Electronics, Information and Bioengineering, Politecnico Di Milano, Piazza Leonardo da Vinci 32, 20133 Milan, Italy

**Keywords:** Long-COVID, SARS-CoV-2, Neurophysiology, Pain, Fatigue, Electromyography

## Abstract

**Introduction:**

Neurological sequelae following SARS-CoV-2 infection still represent a serious concern both for neurologists and neuroscientists. In our paper, we investigated pain, myalgia, and fatigue as symptoms in long-COVID patients with an electrophysiological approach, comprising the evaluation of sympathetic skin responses (SSRs) and quantitative electromyography (qEMG).

**Materials and methods:**

Twelve patients were enrolled (mean age, 47.7 ± 11.6 years), referred to our attention because of myalgia, pain, or muscle cramps, which persisted about 6 months after the diagnosis of SARS-CoV-2 infection. They underwent conventional electroneurography (ENG), needle electromyography (EMG), and SSRs; moreover, qEMG was performed by sampling at least 20 motor unit potentials (20–30 MUPs) during weak voluntary contraction in deltoid and tibialis anterior muscles. The mean duration, amplitude, and percentage of polyphasic potentials were assessed and compared with healthy and age-matched volunteers.

**Results:**

ENG did not disclose significant changes compared to healthy subjects; needle EMG did not reveal denervation activity. In addition, qEMG showed MUPs similar to those recorded in healthy volunteers in terms of polyphasia (deltoid: *p* = 0.24; TA: *p* = 0.35), MUP area (deltoid: *p* = 0.45; TA: *p* = 0.44), mean duration (deltoid: *p* = 0.06; TA: *p* = 0.45), and amplitude (deltoid: *p* = 0.27; TA: *p* = 0.63). SSRs were not recordable from lower limbs in seven patients (58%) and from the upper ones in three of them (25%).

**Conclusion:**

Our data suggest an involvement of the autonomic system, with a focus on cholinergic efferent sympathetic activity, without any evidence of myopathic changes.

## Introduction

It is well known that SARS-CoV-2 leads to several neurological manifestations, including large-vessel strokes, seizures at onset, muscle pain, and para-infectious neuropathies. The neurological involvement is due to different mechanisms of action, probably comprising a direct spread through nerve terminals, as well as a disruption of the blood–brain barrier (BBB), as recently suggested by neuropathological studies in humans [1–4]. Moreover, following the acute phase of the illness, both neurological and non-neurological sequelae occur frequently: they are usually defined as “long-COVID syndrome” and comprise a number of heterogeneous complications, ranging from pain to smell disturbances and cognitive impairment, especially in the domains of processing speed and long-term visuo-spatial and verbal memory [5]. These complications often last for months, and their duration does not correlate with the severity of the primary infection [6].

Among these, recent papers have focused the attention on pain, fatigue, and muscle symptoms, suggesting a combination of central and peripheral mechanisms and highlighting the role of a reduced intracortical GABAergic inhibition underlying their occurrence [7]. Nonetheless, little is known about the neurophysiology of muscle and peripheral nerve involvement in long-COVID patients [8–10]; in particular, it is not clear whether pain, a frequent complication of SARS-CoV-2 infection, is related to the impairment of either peripheral nerve terminals or muscle fibers. Such information could significantly impact not only the prognosis but also the choice of pharmacological and non-pharmacological treatments, guiding rehabilitative interventions, which are still unspecific and refer to protocols used for long-COVID–like symptoms [11].

In this paper, we neurophysiologically evaluated patients with “long-COVID syndrome” referred to our laboratory by performing both qualitative and quantitative electromyography (EMG) and assessing small fiber involvement.

## Materials and methods

### Patients and clinical assessment

Twelve patients (mean age, 47.7 ± 11.6 years; eight women) were enrolled in the study (Table 1). The recruitment took place during the outpatient activity, and each patient was referred to our hospital because of myalgia, pain, or muscle cramps, which persisted about 6 months after the diagnosis of SARS-CoV-2 infection (mean duration of symptoms from the infection, 175.3 ± 31.0 days). Diagnosis of COVID-19 was confirmed by positive results on a reverse-transcriptase-polymerase-chain-reaction (RT-PCR) assay performed on nasopharyngeal and throat swab. None of them was hospitalized because of COVID-19, and only three patients were treated with low-dosage steroids during the acute phase of the illness. None of them reported the assumption of drugs interfering with the neuromuscular transmission at the time of the neurophysiological assessment. Common causes of neuropathies were also excluded by a preliminary interview (i.e., diabetes, dysthyroidism, vitamin B12 and/or folate deficiency, neoplastic/paraneoplastic syndromes). Other exclusion criteria were a pre‐existing neuromuscular transmission failure, polyradiculoneuropathies, and central nervous system disorders.

**Table 1:** Demographic and clinical features of long-COVID patients

ID	Age/sex	Hospitalization	Doses of COVID vaccine	Duration of positivity (days) and symptoms of COVID-19	Time from positivity to ENG/EMG (days)	Sensory symptoms	Muscle symptoms
*1*	34/F	None	**3**	17/fever, cough	155	Paresthesia in hands and feet	Myalgia, fatigue
*2*	27/F	None	**2**	14/fever, cough, and hyposmia	166	Paresthesia in hands	Fatigue
*3*	53/F	None	**3**	12/fever, anosmia, and ageusia	208	Short bursts of pain in arms (especially on the right side)	Myalgia, fatigue
*4*	64/F	None	**3**	10/myalgia and cough	184	Short bursts of burning sensation in arms and feet	Myalgia, fatigue
*5*	48/F	None	**2**	18/fever, anosmia, and ageusia	180	Burning sensation in feet	Myalgia, fatigue
*6*	56/F	None	**2**	8/cough and anosmia	206	Short bursts of burning sensation in arms	Fatigue
*7*	62/M	None	**3**	11/fever and cough	122	Occasional paresthesia in feet	None
*8*	55/M	None	**3**	13/fever, anosmia, and ageusia	225	Paresthesia in feet (especially on the left side)	Fatigue
*9*	34/F	None	**3**	15/cough and anosmia	163	Burning sensation in feet	Myalgia
*10*	49/F	None	**3**	10/fever	144	Paresthesia in hands and feet	None
*11*	41/M	None	**2**	10/fever, hyposmia, and hypogeusia	148	Paresthesia in feet	Myalgia
*12*	50/M	None	**3**	9/cough and hyposmia	203	Occasional paresthesia in feet	None

List of abbreviations. ID: identification number; ENG/EMG: Electroneurography/Electromyography; M. male; F: female

Each patient was asked about physical fatigue, myalgia, joint pain, and muscle cramps. Both sensory and muscle symptoms are reported in Table 1. All patients were vaccinated before entering the study, with either two or three doses (Table 1).

Creatine-kinase (CK) serum levels were within normal limits in all patients (132 ± 44.7 U/L; normal values, < 210 U/L).

### Electroneurography (ENG) and conventional electromyography (EMG)

Neurophysiological parameters were compared with those recorded from 15 healthy and age-matched volunteers (10 women, 49.3 ± 8.9 years). Each patient underwent nerve conduction studies, including a bilateral examination of the median, ulnar, deep peroneal (from the extensor digitorum brevis muscle, EDB) and tibial (abductor hallucis brevis, AH) motor nerves (compound motor action potentials, CMAPs) and of the median, ulnar, radial, and sural sensory nerves (sensory action potentials, SAPs). Abductor digiti minimi muscle (ADM) responses to 3 Hz repetitive stimulation of the ulnar nerve were performed to rule out a neuromuscular transmission disease, at rest (*T*_0_), immediately after 60″ contraction (*T*_1_), 1 (*T*_2_), and 3 min (*T*_3_) later. Changes in CMAP sizes are quantified by calculating the percentage change in negative peak amplitude between the first and the fourth responses.

Electromyographic signal was captured by needle electrodes and analyzed (band-pass 10 Hz–10 kHz; sampling rate, 5 kHz; sensitivity set, 200 µV/Div; sweep speed, 10 ms/Div).

### Evaluation of small fibers

Sympathetic skin responses (SSRs) were evaluated in all patients and recorded from the most affected side, either left or right, then compared with those obtained from 15-age- and sex-matched volunteers.

The stimulator was placed over a median nerve at the wrist, and the stimulation intensity was set at 20 mA (duration, 0.2 ms). Surface electrodes were positioned on palms and soles with reference electrodes placed over the dorsal region [12]. The filter was set at 0.5–2000 Hz. For each limb, three recordings were performed with an inter-stimulus interval between 20 and 30 s to avoid habituation. The response was considered not recordable when no change larger than 50 mV was observed in any of the three recordings in 2 s following a stimulus [12, 13].

### Quantitative electromyography (qEMG)

Qualitative electromyography (EMG) of both deltoid and anterior tibial muscles was performed in healthy controls and patients. Additionally, EMG of other muscles was performed in patients with relevant symptoms [8]. EMG was performed using a concentric 35-mm Dantec needle electrode and the Department́ standard filter settings of 20 Hz–10 kHz, gain (100 mV/division), and sweep speed (10 ms/division). The presence of either denervation (fibrillation potentials and positive sharp waves) or spontaneous activity (e.g., fasciculations) was assessed at three separate sites per muscle, and spontaneous activity at more than two sites was required for abnormality [14]. Rest activity was monitored for at least 60″ at each insertion point.

Quantitative motor unit potential (MUP) analysis was done by sampling at least 20 MUPs (20–30 MUPs) during weak voluntary contraction. The method for multi-MUPs analysis is described in detail elsewhere [15–17]. In brief, the operator determines when the computer is to analyze about 30 s of a previously acquired signal. MUPs are selected and extracted (signal decomposition) according to software definitions and then analyzed by using different algorithms. MUPs are then sorted into classes, each representing the average of the selected MUPs.

The mean duration, amplitude, and percentage of polyphasic (i.e., five phases or more) potentials were assessed [17]. A mean MUP duration lower than the 95% confidence interval of healthy controls was considered as myopathic. This method is now considered the most reliable, fastest, and easiest to perform among three different quantitative EMG methods proposed so far; it usually requires about 5 min per muscle to be performed [18].

EMG interpretation was completed by 2 trained neurophysiologists (T.B.; L.C.), and qEMG was performed on the most affected side, either left or right.

The whole electrophysiological protocol required about 45 min for each patient.

### Statistical analysis

Parametric analyses were used, as all datasets passed the Shapiro–Wilk test for normality (*p* > 0.05). Neurophysiological data were compared between patients and age-matched volunteers by using an unpaired t-test (*p* < 0.05). Data were analyzed using SPSS v. 21.0 for Windows (SPSS Inc.).

## Results

### Electroneurography (ENG) and conventional electromyography (EMG)

ENG and EMG were unremarkable in each patient. None of them showed spontaneous activity (i.e., fibrillation potentials) in any muscle (Table 2), and voluntary contraction showed an interference pattern of activation. Repetitive nerve stimulation (RNS) showed only a mild decrease of CMAP amplitudes until *T*_2_ (*T*_0_: − 3.4 ± 2.1; *T*_1_: − 5.1 ± 1.6; *T*_2_: − 2.5 ± 0.9; *T*_3:_ 4.7 ± 3.3), falling within the normal range as extensively reported in literature [19, 20].

**Table 2:** Electrophysiological findings in healthy controls and patients, as assessed by both conventional and quantitative (MMUA) EMG

	Healthy controls (*N* = 15)	Patients (*N* = 12)	*P* value
*MUP duration (ms) (D)*	10.4 ± 1.8	9.1 ± 1.4	0.06
*MUP amplitude (ΜV) (D)*	528.3 ± 103.5	574.7 ± 106.0	0.27
*MUP area (ΜV/ms) (D)*	839.1 ± 198.2	902.9 ± 235.7	0.45
*Polyphasia % (D)*	6.9 ± 2.6	8.6 ± 4.7	0.24
*MUP duration ( ms) (TA)*	9.3 ± 0.8	9.0 ± 1.7	0.55
*MUP amplitude (ΜV) (TA)*	603.4 ± 351.7	665.0 ± 290.2	0.63
*MUP area (ΜV/ ms) (TA)*	938.5 ± 382.6	1069.9 ± 514.0	0.44
*Polyphasia % (TA)*	6.4 ± 4.5	7.9 ± 3.4	0.35
*Ulnar SCV (m/s)*	54.1 ± 7.7	56.0 ± 6.1	0.49
*Ulnar snap amplitude (ΜV)*	30.7 ± 13.3	32.1 ± 15.7	0.80
*Ulnar MCV (m/s)*	61.1 ± 7.9	59.5 ± 6.1	0.57
*Ulnar CMAP amplitude (mV)*	17.5 ± 4.8	16.8 ± 3.0	0.66
*Peroneal MCV (m/s)*	52.8 ± 2.2	50.9 ± 3.7	0.11
*Peroneal CMAP amplitude (mV)*	11.9 ± 1.8	10.5 ± 3.7	0.21
*Sural SCV (m/s)*	55.1 ± 2.2	53.8 ± 4.0	0.07
*Sural snap amplitude (ΜV)*	13.4 ± 5.1	12.2 ± 7.7	0.63

All values are expressed as mean ± standard deviation. *D*, deltoid; *TA*, tibialis anterior; *MUP*, motor unit potentials; *SCV*, sensory conduction velocity (m/s); *MCV*, motor conduction velocity (m/s); *CMAP*, compound muscle action potentials; *mV*, millivolts; *ms*, milliseconds; *ΜV*, microvolts. Both ulnar and peroneal amplitudes refer to distal CMAPs

### Small fiber involvement

**Fig. 1:**
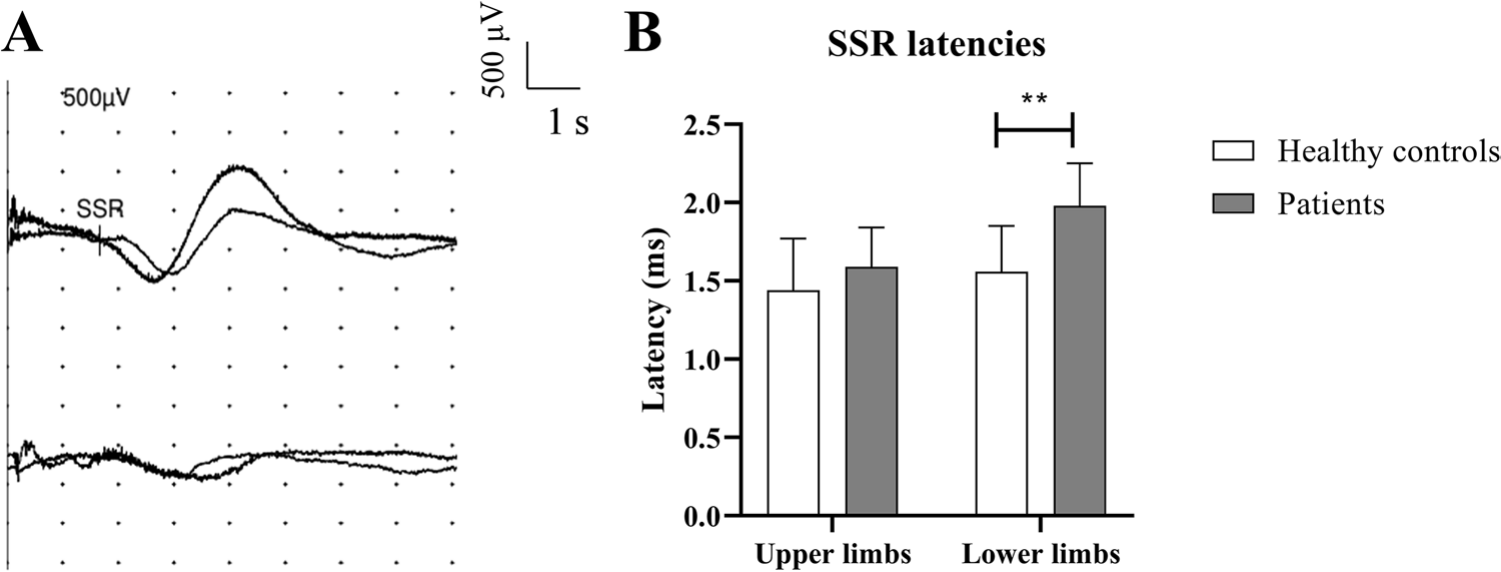
Neurophysiological evidence of a small-fibers neuropathy (SFN). **A** Sympathetic skin responses (SSRs) in a representative patient. Responses were recorded from the lower and the upper limbs at the right side. Note that both responses were not recordable. Responses were obtained by stimulating the wrist with the intensity set at 20 mA. **B** Histogram showing significant differences in terms of SSR latencies between patients and controls (data are reported as mean values ± S.D. ** *p* < 0.01)

SSRs were not recordable from lower limbs in seven patients (58%) and also from the upper ones in four of them (25%; patients 1, 4, 5, and 11, as reported in Table 1). Figure 1A shows a sample of SSRs recorded in a young woman (patient 3).

In remaining cases, SSR latencies did not change between patients and controls when recorded from the upper limbs (1.59 ± 0.25 s versus 1.44 ± 0.33 s: *p* = 0.27), whereas they are significantly longer compared to healthy volunteers when recorded from feet (1.98 ± 0.27 s versus 1.56 ± 0.29 s: *p* = 0.0019, unpaired *t*-test with Welch’s correction; Fig. 1B).

### Quantitative electromyography (MMUA)

**Fig. 2:**
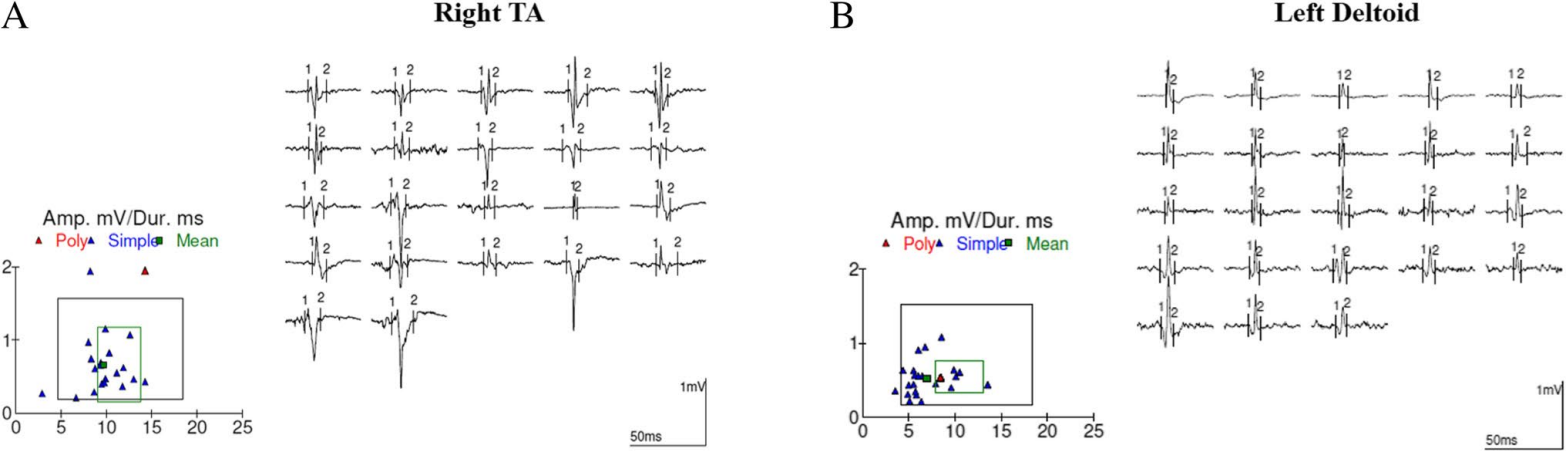
Quantitative EMG in a representative long-COVID patient. **A** Multi-motor unit analysis (MMUA) was performed on tibialis anterior (TA) muscle, on the right side, collecting 22 different MUPs. A Cartesian graph on the left (bottom) shows the distribution of each MUP in terms of amplitude (*y*-axis, expressed as mV) and duration (*x*-axis, ms). **B** MMUA was performed on deltoid muscle, on the left side, collecting 23 different MUPs. A Cartesian graph on the left (bottom) shows the distribution of each MUP in terms of amplitude (*y*-axis, expressed as mV) and duration (*x*-axis, ms). Note that almost all MUPs are within the normal range, and traces show MUPs with a typical triphasic appearance in both muscles

MMUA parameters did not significantly differ between healthy controls and patients (Table 2); in particular, both in deltoid and TA muscle, the percentage of polyphasia (D: *p* = 0.24; TA: *p* = 0.35), MUP area (*p* = 0.45; *p* = 0.44), amplitude (*p* = 0.27; *p* = 0.63), and mean duration (*p* = 0.06; *p* = 0.45) did not change between the two groups. Figure 2 shows MUPs recorded from both deltoid and TA muscle in a representative patient (female, patient 3).

## Discussion

Our data suggest a predominant involvement of the autonomic system, with a focus on cholinergic efferent sympathetic activity, in patients with pain and “long-COVID syndrome,” without any evidence of myopathic changes. There is no correlation between disease severity and electrophysiological findings. To the best of our knowledge, this is the first report combining a neurophysiological evaluation of small fibers with quantitative electromyography (qEMG). Our findings could also contribute to understand pathogenetic mechanisms underlying SARS-CoV-2 neurotropism, possibly guiding both pharmacological and non-pharmacological treatments for long-COVID syndrome. For example, in absence of a clear etiopathogenesis, rehabilitative protocols currently refer to treatments for pathologies with similar clinical presentation, i.e., fibromyalgia or chronic fatigue syndrome, which have a different pathogenesis and underpinning mechanisms [11].

Our results are in line with other studies pointing to a predominant involvement of small fibers in long-COVID [21, 22] and fit with neurophysiological and histopathological data coming from hospitalized patients [1, 23]. Oaklander and colleagues described skin biopsies in long-COVID and confirmed a predominant neuropathic involvement [21]; the hypothesis of an underlying small-fiber pathology is also corroborated by the observation of small-fiber loss applying in vivo corneal confocal microscopy [24]. Also, Abrams and co-workers found changes in skin biopsies supporting the hypothesis of a small fiber neuropathy (SFN) [25].

In a more recent study, SSRs have been assessed in 70 patients, who recovered from COVID-19, showing significantly longer latencies in patients when compared to the control group; although the temporal relationship with the primary infection has not been reported in detail, the authors concluded that SFN can predict disease severity, as well as clinical outcomes and prognosis [26].

Nonetheless, one study reported predominant myopathic features in long-COVID patients, using a neurophysiological protocol similar to that we applied here [8]. The authors based their conclusions on MUP duration and polyphasia found in tibialis anterior (TA) muscle; no changes in terms of MUP amplitude, duration, number of turns, and polyphasia were additionally found in deltoid muscle. Moreover, authors did not perform muscle biopsies, although another study has recently corroborated these findings by skeletal muscle histopathology [27]. Differences between these results and ours may be additionally related to other factors: In some cases, their patients had been hospitalized during the acute phase of the illness, and they were older than ours at the time of first electrophysiological evaluation. Moreover, time elapsed from the infection, and the electrophysiological assessment was longer in our sample. Finally, Agergaard and co-workers did not clarify whether and which myotoxic drugs had been administered during the acute phase, including chloroquine, steroids, ritonavir, or lopinavir.

Overall, it is worth considering that different neurophysiological observations may be explained, at least in part, by different viral variants, possibly triggering different acute and chronic neurological manifestations.

In our sample, patients showing an autonomic involvement were treated with steroids (prednisone; starting dose, 25 mg/day), with a subjective improvement of both sensory and muscle symptoms.

Although our neurophysiological data are strongly corroborated by histopathological findings from other laboratories, they have some limitations. First, we did not perform a skin biopsy in our patients. Second, there is no follow-up of our neurophysiological findings, except for one patient. Third, autonomic changes were assessed by evaluating SSRs only; we did not evaluate the patients for the presence of autonomic symptoms, such as impaired sweating or fluctuations in blood pressure and the heart rate. Equally important, laser-evoked potentials (LEPs) were not assessed. LEPs represent the most sensitive test for the diagnosis of SFN; they allow to evaluate both A-delta and C fibers, highlighting at the same time possible changes at a cortical level, including primary sensory and cingulate cortices [28]. Finally, fatigue and pain should not only be attributed to involvement of the small fibers; they could also be related to central nervous system (CNS) involvement, cardiac involvement, kidney failure, lung disease or anxiety, and depression [29, 30].

## Data Availability

The corresponding author has full access to data and has the right to publish such data. Data will be available upon reasonable request to the corresponding author.
